# SABCS 2017 pathology: from bench to bedside

**DOI:** 10.1007/s12254-018-0427-8

**Published:** 2018-08-15

**Authors:** Zsuzsanna Bago-Horvath

**Affiliations:** Clinical Institute of Pathology, Medical University of Vienna, Währinger Gürtel 18–20, 1090 Vienna, Austria

**Keywords:** SABCS pathology, Molecular pathology, Bisphosphonates, EndoPredict

## Abstract

The 40th International San Antonio Breast Cancer Symposium offered a multifaceted platform for the presentation of several innovative therapeutic approaches. The results of these preclinical and clinical studies provided insight into the development of novel therapy concepts from the laboratory bench to the bedside of breast cancer patients. One main focus of last year’s symposium was the search for synergisms and opportunities for collaboration between basic research scientists and investigators in drug development. Highlights of these topics included preclinical data on selective estrogen receptor covalent antagonists (SERCAs), the discovery of immune-modulating effects of demethylating agents as well as the exact characterization and risk assessment of *BRCA2* mutations of previously unknown significance. Pathological advances aimed at the molecular understanding of intratumoral heterogeneity and the evolution of lobular breast cancer. Beyond preclinical discoveries at the molecular level, clinical studies provided evidence on the duration of adjuvant bisphosphonate treatment and the use of the EndoPredict multigenomic assay to predict response to neoadjuvant chemo- and endocrine therapy. The SUCCESS A study reported that the prolonged adjuvant administration of zoledronic acid for 5 years did not improve patient survival after chemotherapy. A translational analysis of the ABCSG 34 trial revealed that the EndoPredict multigenomic assay could identify patients who do not benefit from neoadjuvant endocrine or chemotherapy. These recent advances are likely to promote individualized breast cancer care.

Personalized medicine presumes comprehensive strategies in drug development that account for novel findings in basic research and utilize newly discovered targets. Several presentations of the 40th San Antonio Breast Cancer Symposium pointed out new possibilities to transfer findings in molecular biology into individualized patient care.

Markus Warmuth, CEO of the biotechnological company H3 Biomedicine (www.h3biomedicine.com) introduced in his talk an innovative reagent that could overcome endocrine resistance caused by mutations in the estrogen receptor (ER). In the course of disease progression, approximately 30% of breast cancers bearing resistance to endocrine therapy acquire mutations of the *ESR1* gene that codes for ER [1]. These mutations result in a constitutive ligand-independent activation of ER that leads to resistance against aromatase inhibitors. To circumvent this problem, a newly engineered substance group named selective estrogen receptor covalent antagonists (SERCAs) was developed. SERCAs bind covalently to ER and effectively block its activation, terminating the signaling cascade. One substance termed H3B-6545 showed promising antitumor activity as a single agent as well as in combination with palbociclib in an animal model.

Fergus Couch from the Mayo Clinic reported about the characterization of *BRCA2* mutations with previously unknown significance [2]. The clinical management of patients with these genetic alterations poses several problems. Since the consequences and outcomes of such mutations are unclear, no recommendations on breast cancer screening and prophylactic operations could be given. Although statistical models for the calculation of a patient’s individual risk exist, there are numerous rare variants with too few affected families that do not allow for an exact assessment of the mutation effects. In these cases, functional assays are likely to provide a viable alternative. In the Mayo Clinic, Couch and colleagues developed a cell-based homology directed repair assay that can test the functionality of the BRCA2 protein. This functionality shows an inverse correlation with the oncogenic potential of the mutated protein. A double-strand DNA break is generated in a green fluorescent protein (GFP) construct, which is then repaired by the BRCA2 protein. If the protein is functional, the cells turn green, indicating a mutation with no oncogenic potential. The assay showed a 100% sensitivity and specificity and could classify most previously problematic mutations. Moreover, using a cell-based chemosensitivity assay, the impact of the variants on the chemosensitivity to platinum compounds could also be investigated.

Besides genetic mutations, epigenetic alterations also play an important role in breast cancer progression and might serve as a target for future therapy approaches [3]. Work done by Cynthia Zahnow and colleagues revealed that tumor cells treated with the demethylating agent 5‑azacytidine demonstrated an upregulation of immune-related genes, some of which were members of the interferon pathway involved in antiviral response [4, 5]. Demethylation seems to reactivate ancient retroviral elements of the genome, which in turn activate the antiviral response. This observation led to the idea of combining inhibitors of the methylating enzyme DNA-N-methyltransferase (DNMT) with an anti-PD-1 antibody. Indeed, the combination of 5‑azacytidine or the DNMT inhibitor givinostat with anti-PD-1 antibodies had synergistic effects in a mouse model.

Jorge Reis-Filho from the Memorial Sloan Kettering Cancer Center highlighted two controversial topics in breast carcinogenesis. The first presentation introduced lobular neoplasia/lobular in situ carcinoma as the molecular precursor lesion of invasive lobular breast cancer. Reis-Filho and colleagues analyzed 43 cases of matched invasive and in situ lobular carcinomas and detected identical mutational landscapes. Consequently, lobular neoplasia/lobular in situ carcinoma could be identified as a nonobligatory precancerous lesion, a precursor of invasive lobular breast cancer [6].

The second presentation addressed the evolution of intratumoral heterogeneity. This phenomenon is present in all types of breast carcinomas and frequently affects common therapy targets such as HER2. Therefore, intratumoral heterogeneity might play a role if therapy resistance develops. Recently, a new technology of single cell genomic sequencing that allows analysis of formalin-fixed paraffin-embedded (FFPE) material was introduced [7]. With the help of this technology the genomic evolution of individual breast cancer cases could be deciphered which pointed to novel findings. These showed that intratumoral heterogeneity applies only to a portion of breast cancer cases. These tumors display two different mutational patterns. Point mutations are usually acquired step by step, whereas gene copy number alterations, which could be detected in in situ lesions (DCIS) as well, arise batch-wise. Further analyses have proven that disseminated tumor cells harbor comparable alterations; however, they might also contain new mutations.

Although these findings are not likely to currently impact the clinical practice of breast cancer treatment, they might influence the development of future therapy options.

One clinical trial that is likely to influence clinical practice was the SUCCESS A study presented by Wolfgang Janni [8]. This phase III trial investigated whether the extended duration of zoledronic acid treatment from 2 to 5 years after adjuvant chemotherapy could increase survival in patients with high risk of breast cancer recurrence. This study was conducted according to a 2 × 2 design: patients were randomized in 2 chemotherapy arms; the control group having been treated by standard anthracyclin- and taxane-containing regimen, whereas gemcitabine was added in the experimental arm. After completing chemotherapy, patients were again randomized to receive zoledronic acid for 2 or 5 years. Although the trial posed some shortcomings such as the low rate of recurrences, it could provide valuable insights into adjuvant zoledronic acid treatment. The extended treatment did not improve disease-free and overall survival in all subgroups analyzed. Moreover, longer zoledronic acid treatment duration was associated with an increased incidence of adverse events: the rate of osteonecroses of the jaw doubled in the experimental arm. A secondary, experimental end point of the trial was the detection of circulating tumor cells 5 years after the end of chemotherapy: the extended zoledronic acid duration did not significantly decrease the number of circulating tumor cells detected.

Although numerous findings on multigenomic assays had been presented at past symposia, this year the only presentation in a plenary session reported about the use of the EndoPredict multigenomic test. Peter Dubsky presented the analysis of the ABCSG 34 neoadjuvant trial on the efficacy of the EndoPredict multigenomic assay to predict response to neoadjuvant endocrine and chemotherapy [9, 10]. This analysis included all patients with estrogen-receptor (ER) positive tumors who received neoadjuvant chemo- or endocrine therapy within the ABCSG 34 trial. Results of the investigation showed that the EndoPredict assay was able to predict therapy response measured by residual cancer burden. In luminal A breast cancer with low proliferation rate, a high EndoPredict score (high risk of recurrence) predicted for inadequate tumor response after neoadjuvant letrozole treatment. Accordingly, in luminal B breast cancer, a low EndoPredict score (low risk of recurrence) identified patients who did not benefit from neoadjuvant chemotherapy. In this therapy arm, all patients with optimal tumor reduction displayed a high EndoPredict score. These results imply that the EndoPredict multigenomic assay might be applied to patient selection in future neoadjuvant trials.

The highlighted presentations constituted only a fraction of novel discoveries presented at the 40th International San Antonio Breast Cancer Symposium, which might enrich breast cancer diagnosis and treatment with new facets.
